# HDAC6 Mediates Poly (I:C)-Induced TBK1 and Akt Phosphorylation in Macrophages

**DOI:** 10.3389/fimmu.2020.01776

**Published:** 2020-08-11

**Authors:** Yan Wang, Ke Wang, Jian Fu

**Affiliations:** ^1^Department of Respiratory and Critical Care Medicine, The Second Hospital of Jilin University, Changchun, China; ^2^Department of Toxicology and Cancer Biology, College of Medicine, University of Kentucky, Lexington, KY, United States

**Keywords:** innate immunity, infection, cytokine, acetylation, microtubule

## Abstract

Macrophages are derived from monocytes in the bone marrow and play an important role in anti-viral innate immune responses. Macrophages produce cytokines such as interferons and IL-10 upon viral infection to modulate anti-viral immune responses. Type I interferons (IFNs) promote anti-viral defense. IL-10 is a suppressor cytokine that down-regulates anti-viral immune responses. HDAC6 is a tubulin deacetylase that can modulate microtubule dynamics and microtubule-mediated cell signaling pathways. In the present study, we investigated the potential role of HDAC6 in macrophage anti-viral responses by examining poly (I:C)-induced IFN-β and IL-10 production in mouse bone marrow-derived macrophages (BMDMs). We also investigated the role of HDAC6 in poly (I:C)-induced anti-viral signaling such as TBK1, GSK-3β, and Akt activation in mouse BMDMs. Our data showed that HDAC6 deletion enhanced poly (I:C)-induced INF-β expression in macrophages by up-regulating TBK1 activity and eliminating the inhibitory regulation of GSK-3β. Furthermore, HDAC6 deletion inhibited poly (I:C)-induced suppressor cytokine IL-10 production in the BMDMs, which was associated with the inhibition of Akt activation. Our results suggest that HDAC6 modulates IFN-β and IL-10 production in macrophages through its regulation of TBK1, GSK-3β, and Akt signaling. HDAC6 could act as a suppressor of anti-viral innate immune responses in macrophages.

## Introduction

Viral infection is a major health burden worldwide (1–3). The innate immune system, which is at the forefront against viral infection, is required to orchestrate ant-viral immune responses (4–6). Macrophages play a critical role in anti-viral innate immune responses (7–11). Macrophages are derived from monocytes in the bone marrow (12). The monocytes differentiate into macrophages after entering the tissues through circulation (12). Macrophages are activated during viral infection to launch anti-viral defense and to eliminate viral pathogens (7–10). Cytokines, chemokines, and anti-viral proteins produced by macrophages are needed for effective anti-viral defense (7–10).

Type I interferons (IFNs) play an important role in anti-viral innate immune responses by up-regulating anti-viral defense (13, 14). Type I IFNs contain several subtypes such as IFN-α and IFN-β (13, 14). Their expression is dependent on the stimuli and cell types (13, 14). The expression of Type I IFNs is regulated at the transcriptional level by IFN regulatory factors (IRFs) (13, 14). IL-10 is a suppressor cytokine during viral infection (15, 16). IL-10 has been reported to down-regulate anti-viral immune responses and delay virus elimination (15, 16). Macrophages are mighty anti-viral effector cells of innate immunity (7–10). However, macrophages are also a major source of IL-10 production during viral infection (15, 16). Viral infection could induce IL-10 production in macrophages (15, 16). The increased suppressor cytokine IL-10 could then suppress anti-viral responses such as the type I IFN expression and allow the escape of virus from the immune defense (15, 16). Therefore, the timing and balance of IL-10 and Type I IFN expression could be important to control viral infection (15, 16).

TANK-binding kinase 1 (TBK1), an IKK-related serine/threonine kinase, is a critical player in anti-viral immunity (17, 18). TBK1 regulates anti-viral type I interferon production (17, 18). TBK1 activation by viral components such as double stranded RNA (dsRNA) binding to TLR3 leads to signaling transduction that induces type I IFN expression (17, 18). The activity of TBK1 is regulated by TBK1 phosphorylation (17, 18). Glycogen Synthase Kinase-3β (GSK-3β) is a key modulator of TBK1 activity (17–19). GSK-3β binds to TBK1 and induces TBK1 phosphorylation upon viral infection (17–19). Several studies suggest that there are close interactions between GSK-3β and microtubules (20–24). HDAC6, a unique cytoplasmic class II deacetylase, is a well-established tubulin deacetylase that can modulate microtubule dynamics and microtubule-mediated cellular responses (25–27). However, the role of HDAC6 in anti-viral signaling and responses in macrophages remains largely unknown.

Polyinosinic-polycytidylic acid (Poly (I:C)), a synthetic analog of viral double stranded RNA (dsRNA) (28, 29), has been used as a dsRNA ligand of TLR3 to simulate viral infection (28, 29). TLR3 is expressed in many immune cells including macrophages (7–9, 29). Binding of poly (I:C) to TLR3 activates cell signaling pathways and induces type-1 interferon (IFN) responses (28, 29). In the present study, we investigated HDAC6 regulation of poly (I:C)-induced TBK1 activation, GSK-3β phosphorylation, and IFN-β expression in bone marrow-derived macrophages. Furthermore, AKT, which can modulate IL-10 production and GSK-3β activity (30, 31), has been reported to interact with microtubules (32, 33). In our studies, we also examined the role of HDAC6 in poly (I:C)-induced AKT phosphorylation and IL-10 production in macrophages.

## Methods

### Reagents

GSK-3β (Cat#12456), β-actin (Cat#5125), GAPDH (Cat#8884), AKT (Cat#4691), TBK1 (Cat#3504), phospho-TBK1 (Ser172) (Cat#5483), phospho-AKT (ser473) (Cat#4060), HDAC6 (Cat#7612), phospho-GSK3β (Cat#9323), and acetyl-α-tubulin (Lys40) (Cat#5335) antibodies were purchased from Cell Signaling Technology (Danvers, Massachusetts). Poly (I:C) was purchased from Sigma Aldrich (Cat#P9582; St. Louis, Missouri). M-CSF was obtained from R&D Systems (Cat#416-ML; Minneapolis, Minnesota).

### Mouse Bone Marrow-Derived Macrophages

Wild type C57BL/6J mice and HDAC6 knockout C57BL/6J mice were obtained from The Jackson Laboratory (Bar Harbor, Maine). HDAC6 deletion in C57BL/6J mice is produced by CRISPR/Cas9-generated HDAC6 gene knock-out mutation. 9- to 15-week-old sex and age-matched HDAC6 knockout C57BL/6J mice and wild type C57BL/6J mice were used for the studies. All experiments and animal care procedures were approved by the Institutional Animal Care and Use Committee of the University of Kentucky. Mouse bone marrow-derived macrophages were prepared as described preciously (34). Briefly, bone marrow cells were flushed out from femurs and tibias of mice with cold DMEM medium containing 0.5 mM EDTA. The cell suspension was then passed through a cell strainer, centrifuged, and resuspended in DMEM medium containing 10% FBS and 20 ng/ml M-CSF. The cells were seeded into a cell culture plate and maintained in an incubator with 5% CO_2_ at 37°C for 5 d with fresh medium added every other day. After day 5, fully differentiated cells were stimulated with poly (I:C).

### Immunoblotting and ELISA Assays

Immunoblotting assays were performed as described previously (35, 36). Briefly, the protein samples were separated by SDS-PAGE electrophoresis and transferred to polyvinylidenedifluoride membranes. After probing with primary and secondary antibodies, the membranes were developed using a Clarity Western ECL Substrate (Cat#1705061; Bio-Rad, Hercules, California). IFN-β and IL-10 levels in the cell culture supernatant of BMDMs were examined by the mouse IFN-β ELISA kit (Cat# 42410; PBL Assay Science; Piscataway, New Jersey) and mouse IL-10 ELISA kit (Cat#431414; Biolegend; San Diego, California) according to the manufacturer's protocol.

### Statistical Analysis

All experiments were repeated three times. Data are expressed as mean ± SEM. The Student's *t*-test was used for comparisons of two groups. ANOVA and *post hoc* multiple comparison tests were employed for multiple groups. Statistical significance was assigned to a *P* < 0.05.

## Results

**Figure 1:**
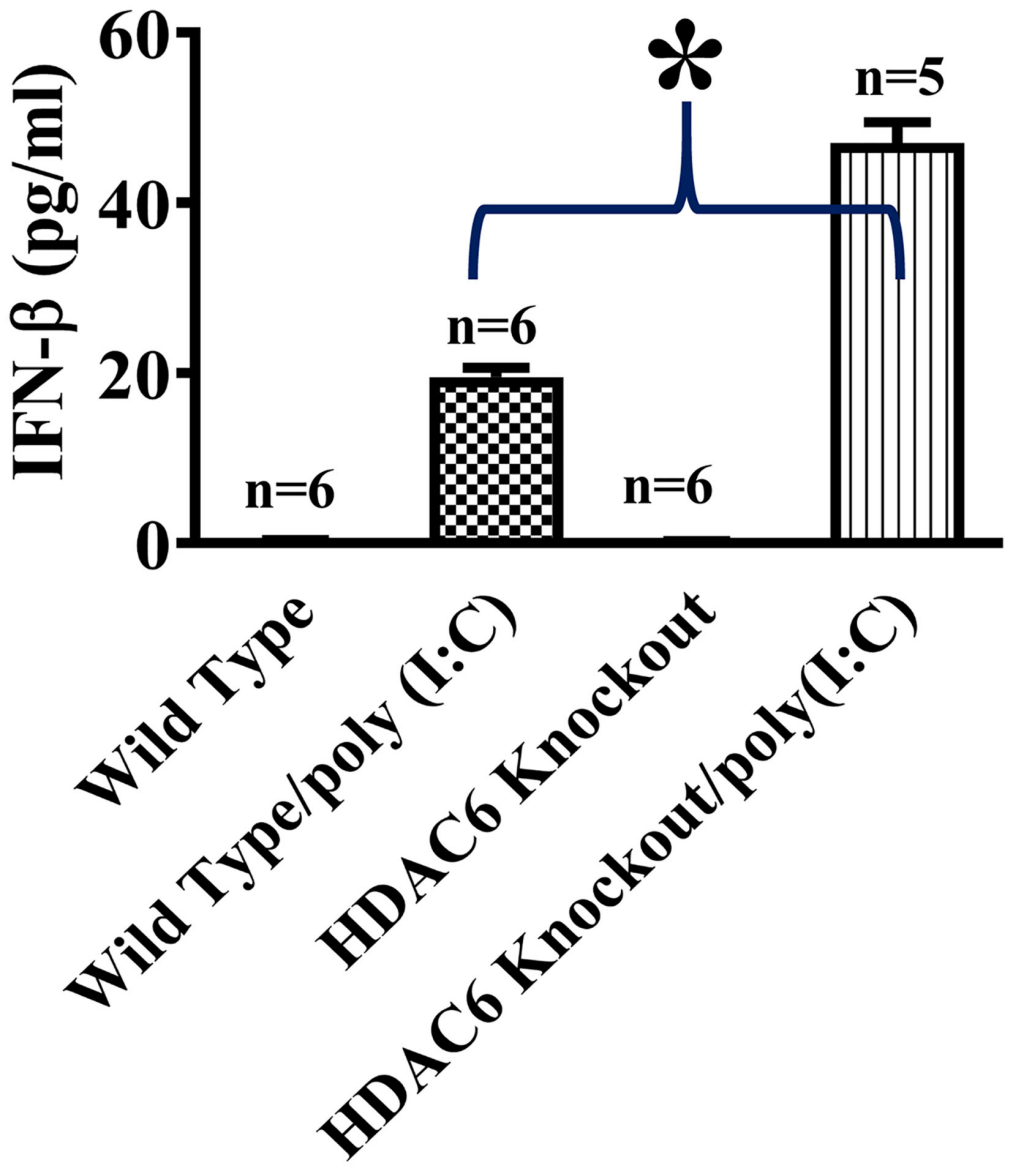
HDAC6 deletion enhances poly (I:C)-induced IFN-β production in BMDMs. BMDMs from the HDAC6 knockout and wild type mice (300,000 cells/well) were challenged without or with 100 μg/ml poly (I:C) for 12 h. Experiments were repeated three times. IFN-β production was assessed by ELISA. *P < 0.05.

To investigate the role of HDAC6 in poly (I:C)-induced type I IFN responses, we first examined the effects of HDAC6 deletion on poly (I:C)-induced IFN-β production in macrophages. BMDMs from the HDAC6 knockout and wild type mice were challenged with poly (I:C). Our data showed that HDAC6 deletion markedly enhanced poly (I:C)-induced IFN-β production in the BMDMs (Figure 1).

**Figure 2:**
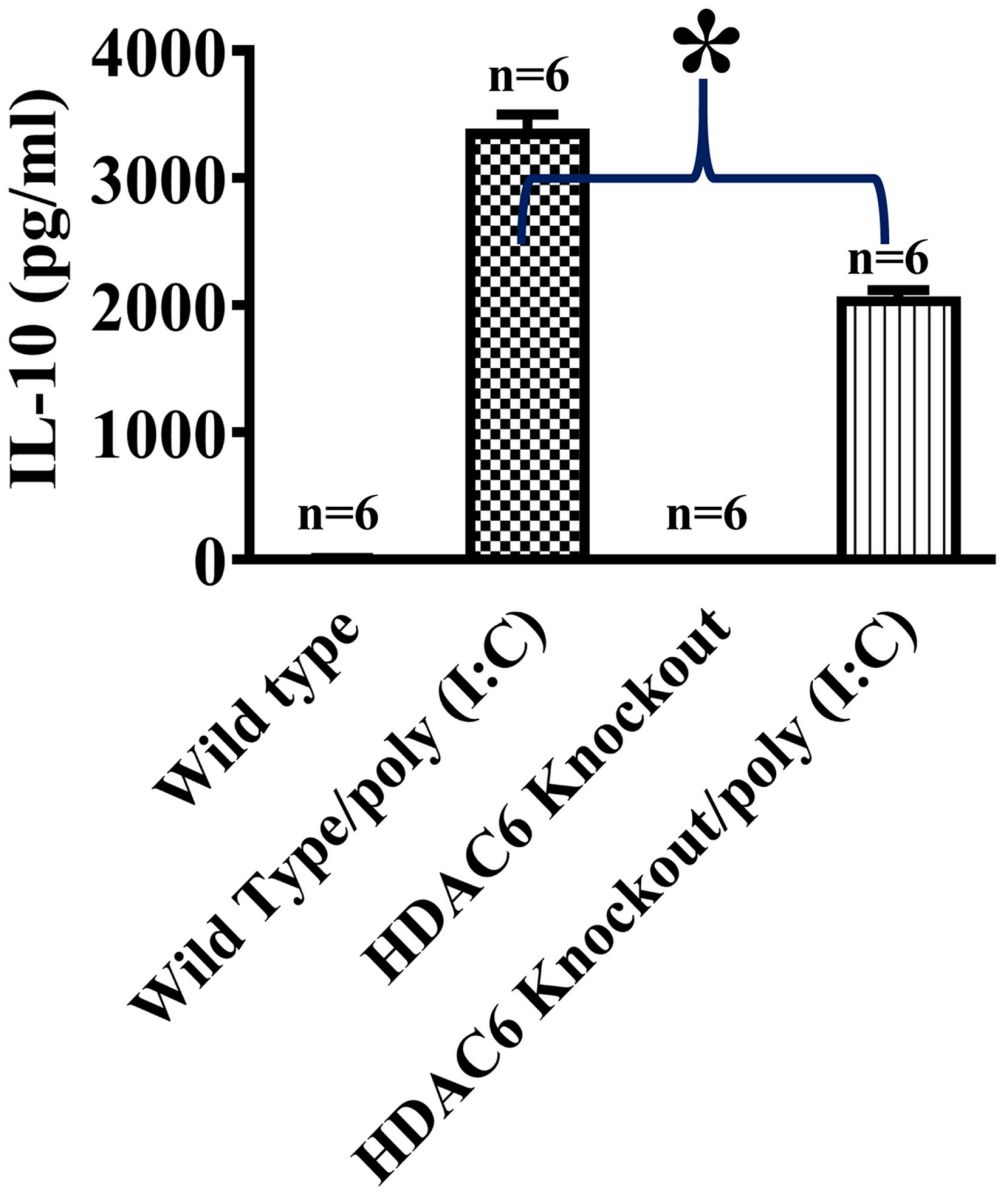
HDAC6 deletion suppresses poly (I:C)-induced IL-10 production in BMDMs. BMDMs from the HDAC6 knockout and wild type mice (300,000 cells/well) were challenged without or with 100 μg/ml poly (I:C) for 12 h. Experiments were repeated three times. IL-10 production was assessed by ELISA. *P < 0.05.

We then conducted experiments to assess the role of HDAC6 in poly (I:C)-induced suppressor cytokine IL-10 production in macrophages. BMDMs from the HDAC6 knockout and wild type mice were challenged with poly (I:C). Our data showed that HDAC6 deletion inhibited poly (I:C)-induced IL-10 production in the BMDMs (Figure 2).

**Figure 3:**
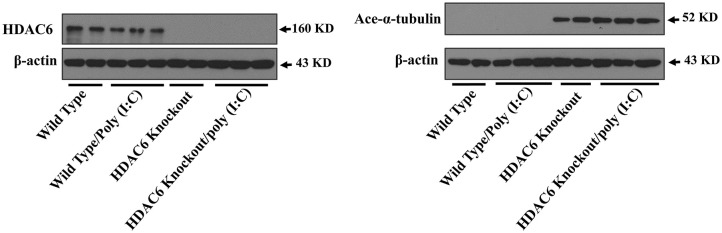
HDAC6 deletion induces robust α-tubulin acetylation in BMDMs. BMDMs from the HDAC6 knockout and wild type mice were challenged without or with 20 μg/ml poly (I:C) for 30 min. Experiments were repeated three times. Representative blots of HDAC6 expression and α-tubulin acetylation (ace-α-tubulin).

Microtubule is involved in modulating a variety of cell signaling pathways (27, 33). Tubulin acetylation status controls microtubule stability and dynamics (27, 33, 37). Tubulin is an endogenous substrate of HDAC6 (25, 27). We conducted immunoblotting assays to examine the effects of HDAC6 deletion on tubulin acetylation status in macrophages. Our results showed that HDAC6 deletion caused hyperacetylation of α-tubulin in the BMDMs (Figure 3).

**Figure 4:**
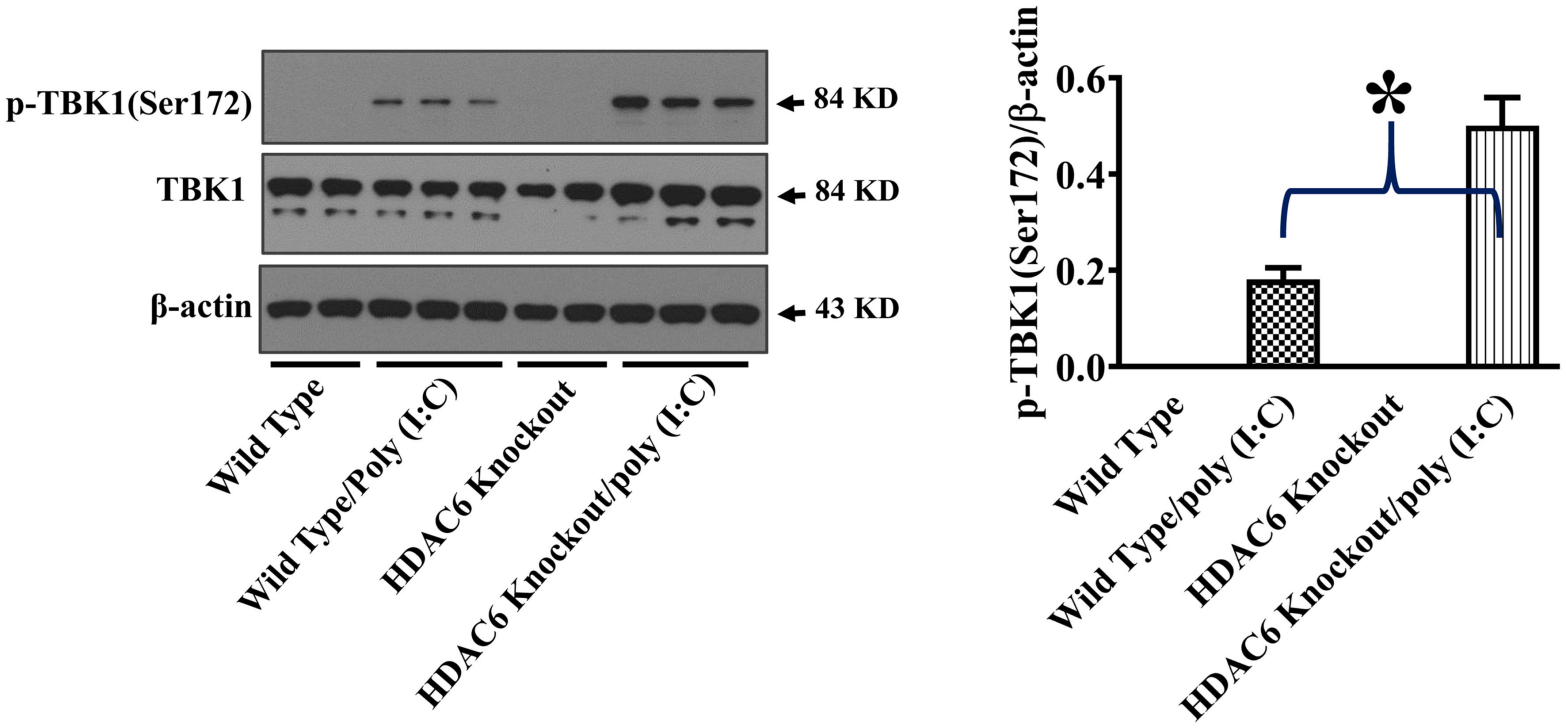
HDAC6 deletion promotes poly (I:C)-induced TBK1 activation in BMDMs. BMDMs from the HDAC6 knockout and wild type mice were challenged without or with 20 μg/ml poly (I:C) for 30 min. Experiments were repeated three times. Representative blots and densitometry analysis of TBK1 phosphorylation at Ser172. *P < 0.05.

TBK1 activation up-regulates type I IFN expression and anti-viral immunity (17, 18). To assess the effects of HDAC6 deletion on anti-viral signaling in macrophages, we examined TBK1 modulation by HDAC6. HDAC6 deletion enhanced poly (I:C)-induced TBK1 activation in the BMDMs as showed by the increased TBK1 phosphorylation at Ser172 of its kinase activation loop (Figure 4).

**Figure 5:**
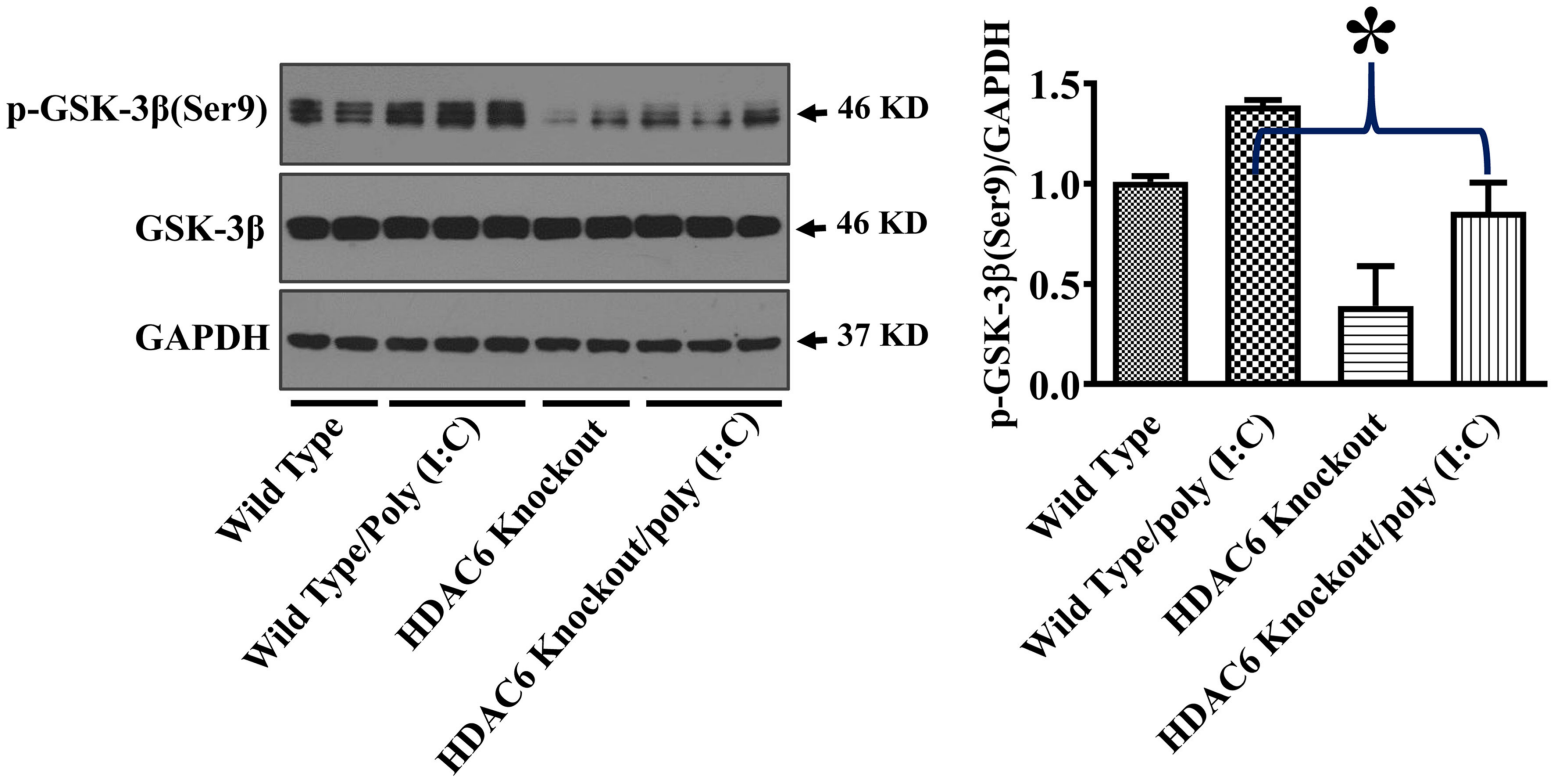
HDAC6 deletion eliminates the inhibitory regulation of GSK-3β in BMDMs. BMDMs from the HDAC6 knockout and wild type mice were challenged without or with 20 μg/ml poly (I:C) for 15 min. Experiments were repeated three times. Representative blots and densitometry analysis of GSK-3β phosphorylation at Ser9. *P < 0.05.

GSK-3β interacts with microtubules and directly binds to TBK1 to modulate TBK1 activity (17–20, 24, 38). GSK-3β function is suppressed by an inhibitory phosphorylation at Ser9 (17–19). To investigate the potential involvement of GSK-3β in HDAC6- and tubulin acetylation-mediated TBK1 modulation, we examined the effects of HDAC6 deletion on GSK-3β phosphorylation at Ser9. Our data showed that HDAC6 deletion led to a marked reduction of GSK-3β phosphorylation at Ser9 in BMDMs (Figure 5), indicating that HDAC6 deletion increases GSK-3β activity.

**Figure 6:**
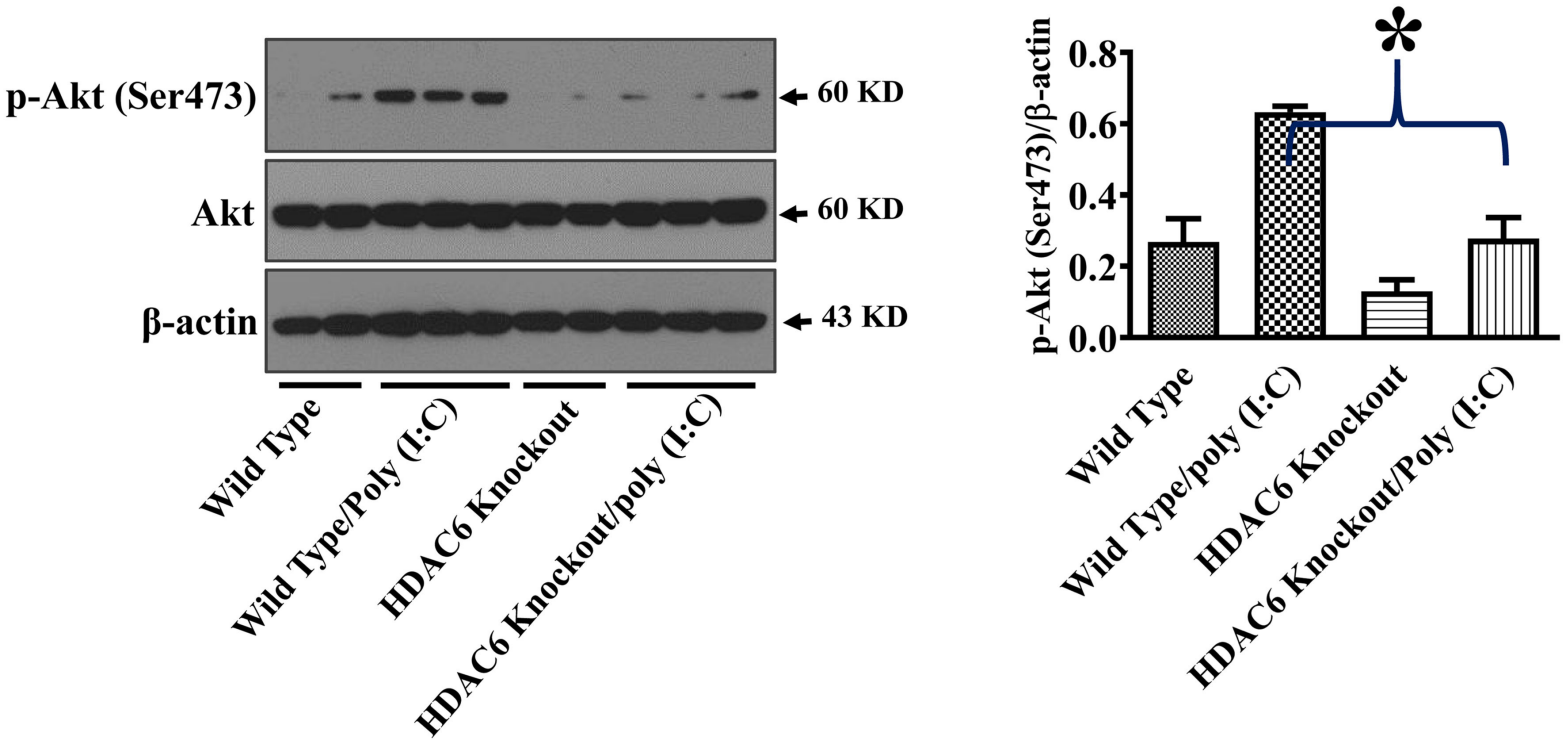
HDAC6 deletion inhibits poly (I:C)-induced Akt activation in BMDMs. BMDMs from the HDAC6 knockout and wild type mice were challenged without or with 20 μg/ml poly (I:C) for 15 min. Experiments were repeated three times. Representative blots and densitometry analysis of Akt phosphorylation at Ser473. *P < 0.05.

Akt is another cell signaling molecule that interacts with microtubules (32, 33). Akt has been reported to modulate IL-10 expression and GSK-3β activity (30, 31). To assess the potential modulation of Akt by HDAC6 and tubulin acetylation, we conducted experiments to examine the effects of HDAC6 deletion on Akt activation by examining Akt phosphorylation at Ser473 that is known to mediate Akt activation. In our studies, HDAC6 deletion inhibited poly (I:C)-induced Akt phosphorylayion at Ser473 (Figure 6).

## Discussion

Macrophages play a central role in defending against viral infection (7–10). We investigated the role of HDAC6 in poly (I:C)-induced anti-viral Type I interferon (IFN) and the suppressor cytokine IL-10 production in bone marrow-derived macrophages. Type I interferon (IFN) responses are fundamental in host innate immunity against viruses (13, 14), whereas the production of IL-10 by macrophages during viral infection has been reported to downregulate type I interferon anti-viral immune responses and hamper virus elimination (15, 16). The interactions of the type I interferon- and IL-10-mediated pathways could play an important role in modulating innate immune responses during viral infection (15, 16). One previous study indicates that HDAC6 could modulate anti-viral responses in different cell types including monocytes and fibroblasts (39). However, *in* our studies, poly (I:C)-induced IFN-β production was increased by HDAC6 deletion in BMDMs, whereas poly (I:C)-induced IL-10 production was inhibited by HDAC6 deletion in BMDMs. Our data suggest that HDAC6 could function as a suppressor of anti-viral responses in macrophages by suppressing anti-viral cytokine IFN-β expression while elevating the suppressor cytokine IL-10 expression.

HDAC6 has been reported to modulate many signaling pathways that could modulate anti-viral immune responses including NF-κB, ERK, and inflammasome activation (27, 40, 41). We examined some key signaling components in anti-viral responses. TBK1 is a key kinase in anti-viral innate immunity that activates the transcription factor IRF3 to induce type I IFN production (18, 19). Autophosphorylation of TBK1 at Ser172 within its kinase activation loop is needed for its activation (18, 19). TBK1-mediated anti-viral immune responses can be regulated by controlling Ser172 autophosphorylation (18, 19). Poly (I:C) induces type 1 interferon (IFN) responses through its binding to TLR3 and activating TBK1 (18, 19, 28). In our studies, HDAC6 deletion led to a marked increase of poly (I:C)-induced TBK1 autophosphorylation at Ser172, which correlates with the increased IFN-β production in the macrophages. Our data indicate that HDAC6 could inhibit TBK1 activity during dsRNA viral infection to suppress type 1 interferon (IFN) responses.

GSK-3β is a major modulator of TBK1 activity (18, 19). GSK-3β binds to TBK1 and enhances TBK1 activity by facilitating its auto-phosphorylation at Ser172 during viral infection, which then up-regulates type I IFN responses (18, 19). Phosphorylation of GSK-3β at Ser9 has been known to inhibit GSK-3β function (30, 42). HDAC6 deletion led to a reduction of the inhibitory GSK3β phosphorylation at Ser9 in macrophages during poly (I:C) challenge, which was associated with the increased TBK1 activation. Our results suggest that HDAC6 can regulate anti-viral responses through GSK-3β. GSK3β has been reported to interact with microtubules (22–24). Our results showed that HDAC6 deletion led to a robust α-tubulin acetylation in macrophages. HDAC6, by modulating tubulin acetylation status, could suppress GSK-3β function and promote GSK-3β Ser9 phosphorylation through its control of microtubule dynamics.

Akt has been reported to modulate IL-10 expression and GSK-3β activity (30, 31). Akt interacts with microtubules, and their interactions are regulated by tubulin acetylation (32, 33). HDAC6 modulates a wide range of cellular responses and signaling through deacetylation of tubulin and microtubules (25–27). HDAC6 deletion inhibited poly (I:C)-induced Akt activation as demonstrated by the reduced Akt phosphorylation within the carboxy terminus at Ser473. Our data suggest that HDAC6 could modulate Akt activation in macrophages during viral infection. The inhibition of Akt activation by HDAC6 deletion could contribute to the decrease of poly (I:C)-induced IL-10 production in the macrophages. Furthermore, Akt-mediated phosphorylation of GSK-3β at Ser9 has been reported to inhibit GSK-3β activity (30). The inhibition of poly (I:C)-induced Akt activation by HDAC6 deletion could also contribute to the elimination of inhibitory phosphorylation of GSK-3β at Ser9.

In summary, our data indicate that HDAC6 deletion enhances poly (I:C)-induced INF-β expression by up-regulating TBK1 activity in macrophages, which is accomplished by eliminating the inhibitory regulation of GSK-3β. Furthermore, HDAC6 deletion inhibits poly (I:C)-induced suppressor cytokine IL-10 production in macrophages, which is associated with the decreased Akt activation. Our results suggest that HDAC6 could act as a suppressor of anti-viral innate immune responses in macrophages.

## Data Availability Statement

All datasets presented in this study are included in the article.

## Ethics Statement

The animal study was reviewed and approved by the Institutional Animal Care and Use Committee of the University of Kentucky.

## Author Contributions

YW designed the study, performed the experiments, edited the manuscript, and analyzed the data. KW designed the study, analyzed the data, and edited the manuscript. JF designed the study, analyzed the data, and wrote the manuscript.

## Conflict of Interest

The authors declare that the research was conducted in the absence of any commercial or financial relationships that could be construed as a potential conflict of interest.
